# Efficacy and safety of direct oral anticoagulants for secondary prevention of cancer associated thrombosis: a meta-analysis of randomized controlled trials

**DOI:** 10.1038/s41598-020-75863-3

**Published:** 2020-11-03

**Authors:** Ruchi Desai, Gautam Krishna Koipallil, Nelson Thomas, Rahul Mhaskar, Nathan Visweshwar, Damian Laber, Ankita Patel, Michael Jaglal

**Affiliations:** 1 Department of Hematology, Morsani College of Medicine, University of South Florida, Tampa, FL 33612 USA; 2 Department of Internal Medicine, Morsani College of Medicine, University of South Florida, Tampa, FL 33612 USA; 3 Division of Hematology, The Moffitt Cancer Center and Research Institute, Tampa, FL 33612 USA; 4 Tampa General, 3 Tampa General Circle, Tampa, FL 33606 USA

**Keywords:** Cancer, Medical research, Oncology

## Abstract

Direct oral anticoagulants (DOACs) may be good alternatives to low molecular weight heparin (LMWH) or vitamin K antagonists (VKA) for treatment of cancer associated thrombosis (CAT). We conducted a meta-analysis of ten randomized clinical trials to evaluate the efficacy and safety of DOACs in patients with CAT. All had study populations composed in entirety or in part of patients with CAT. The primary outcome (efficacy) was recurrent VTE and the secondary outcomes (safety outcomes) included major bleeding, clinically relevant non-major bleeding (CRNMB), and all bleeding (major bleeding + CRNMB). Participants treated with DOACs had lower risk of recurrent VTE, overall (RR 0.63; 95% CI 0.51–0.79; p < 0.0001), compared to LMWH (RR 0.57; 95% CI 0.40–0.83; p = 0.003), but not compared to VKA (RR 0.69; 95% CI 0.44–1.06; p = 0.09). Compared to LMWH, DOACs showed no difference in major bleeding risk (RR 1.31; 95% CI 0.78–2.18; p = 0.31), though had higher risk of CRNMB (RR 1.60; 95% CI 1.13–2.26; p = 0.008) and all bleeding (RR 1.49; 95% CI 1.10–2.01; p = 0.010). These results indicate that DOACs are more effective than LMWH for prevention of recurrent VTE with CAT though carry an increased risk for non-major bleeding compared to standard of care, LMWH.

## Introduction

Active malignancy is a well described prothrombotic state^1^ and cancer patients carry a four to seven fold higher increased risk for venous thromboembolism (VTE) including pulmonary embolism (PE) and deep vein thrombosis (DVT) compared to the general population^2,3^. Cancer associated thrombosis (CAT) is consequential, resulting in significant morbidity and increased mortality^4–6^. Even with anticoagulation, the reported incidence of recurrent VTE can be as high as 6%, or 3.5 times that of the general population with VTE^7,8^.

For the past decade, the standard of care for treatment of CAT has been low molecular weight heparin (LMWH) following the landmark CLOT study demonstrating superiority of dalteparin over vitamin K antagonist (VKA) for prevention of recurrent VTE^9^. However, in clinical practice, providing optimal treatment with LMWH is challenging. Reports show that only half of all patients are fully adherent and many discontinue treatment prematurely^10^. Poor compliance with LMWH occurs for a variety of reasons including financial burden, inconvenience of administration, or development of painful hematoma and scarring at the injection site. Furthermore, adequate dosing of LMWH in elderly patients, patients with impaired renal function, and obese patients is difficult to achieve due to variable absorption, metabolism, and clearance^11^.

Direct oral anticoagulants (DOACs) including dabigatran, rivaroxaban, apixaban, and edoxaban, have less variable pharmacokinetics with rapid onset of action, uniform peak levels, and clearance less affected by extrinsic factors^12^. Given these reasons and ease of administration, DOACs are a desirable alternative to LMWH for patients with active malignancy who may need prolonged anticoagulation. DOACs have demonstrated comparable efficacy and safety to VKA in unselected cancer subpopulations from major RCT^13–18^. Separately, rivaroxaban, apixaban, and edoxaban were shown to be non-inferior to LMWH for secondary VTE prevention in patients with CAT^19–21^. However, clinical guidelines, including those of the National Comprehensive Cancer Network (NCCN) continue to recommend LMWH as the first line treatment for CAT and only recently adopted edoxaban, rivaroxaban and apixaban as treatment options for CAT. Other DOACs are listed as secondary treatment options in patients with compelling reasons to avoid LMWH^22^. Here, we present a meta-analysis to evaluate the clinical usefulness of all DOACs for the treatment of CAT, considering the efficacy and safety of this category of anticoagulants.

## Methods

**Figure 1:**
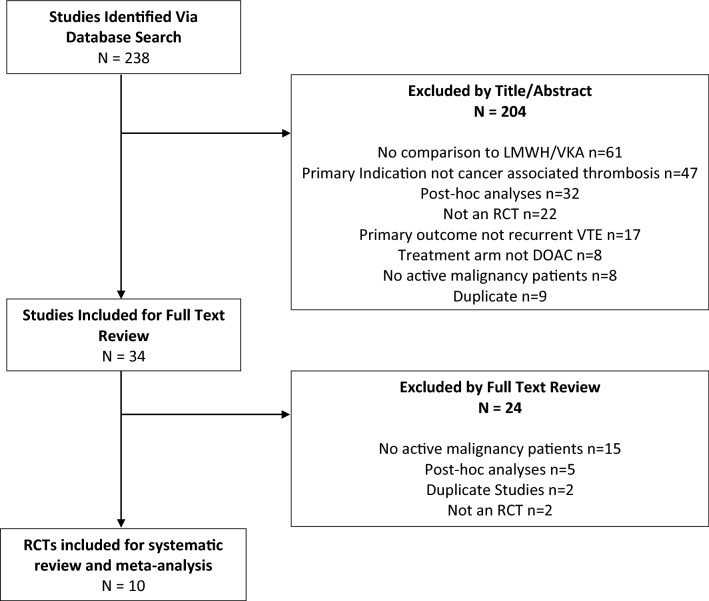
Prisma flow diagram of selection of studies.

The methods for this meta-analysis are in accordance with “Preferred Reporting Items for Systematic Reviews and Meta-Analysis (PRISMA)” (Fig. 1).

### Search strategy

We conducted a systemic literature search of MEDLINE, EMBASE, and Cochrane Central Register of Controlled Trials (CENTRAL) data bases from June 1, 2014-April 31, 2020. We hand searched the American Society for Clinical Oncology (ASCO) and American Society of Hematology (ASH) annual meeting guidelines from 2017–2019. We identified all randomized clinical trials (RCT) which enrolled patients with active malignancy for inclusion in the study for further review. Detailed search strategy is shown in Fig. 1.

### Study selection

Two authors (GK and NT) independently identified studies eligible for inclusion in the systematic review based on screen of titles and abstracts. Discrepancies were resolved by consensus. At all stages of screening, number of studies identified and reasons for inclusion and exclusion were documented. Full papers were included for review if they met inclusion criteria for the meta-analysis. Inclusion criteria were: RCT with study population consisted in whole or in part of adult (age > 18 years) patients with active malignancy and CAT with intervention consisting of DOAC (dabigatran, rivaroxaban, apixaban, or edoxaban) compared to LMWH or VKA.

### Data extraction and quality assessment

Two authors (GK and NT) extracted data independently in duplicate. The primary outcome (efficacy outcome) of interest was incidence of recurrent VTE. The secondary outcomes (safety outcome) of interest was incidence of major bleeding (MB), clinically relevant non-major bleeding (CRNMB), and all bleeding events (composite MB and CRNMB). Outcomes were defined according to criteria used in the included studies though most studies noted bleeding outcomes per ISTH criteria^23^. Data regarding methods, conduct, and design of studies were extracted for assessing risk of bias. We employed the Cochrane Collaboration’s risk of bias tool to assess risk for bias^24^. The authors independently judged quality domains using a two point scale: low risk of bias, plausible bias is unlikely to seriously alter results; high risk of bias, plausible bias that may seriously weaken confidence in results. The GRADE approach was applied to assess quality of evidence for each outcome^25^. We used the GRADE-Pro software to create an evidence profile^26^.

### Statistical analysis

For each outcome, data was pooled using the Mantel–Haenszel method and random effects model was applied to report risk ratio (RR) and 95% confidence interval (CI). In cases where the study authors did not separately report number of MB and CRNMB, bleeding data was applied to all bleeding events. The Cochran χ^2^ test and I^2^ statistic were used to test for heterogeneity between studies. We deemed I^2^ > 50% as substantial heterogeneity. A p-value < 0.05 was considered statistically significant^24^. Two separate subgroup meta-analysis of studies that evaluated DOAC compared to LMWH and DOAC compared to VKA were performed. Overall met-analysis incorporating all studies was also conducted. The meta-analysis was conducted using the Review Manger (RevMan), version 5.3 software^27^.

## Results

### Results of search

The data base search identified 238 citations. 204 citations were excluded by title and abstract alone. Thirty-four studies were evaluated in full text review. Of these, 24 were excluded based on the following reasons: 15 did not include any active cancer patients, five were post-hoc analyses of already included studies, two studies were duplicates, two were not RCT with one study being a risk benefit analysis of an already included study and one study was an economic analysis. We included ten RCTs in this systematic review and meta-analysis (Fig. 1).

### Included studies

We included ten RCTs comparing DOAC to VKA or LMWH for treatment of CAT in patients with active malignancy. One RCT evaluated apixaban compared to enoxaparin followed by warfarin (AMPLIFY)^13^; two RCTs evaluated rivaroxaban compared to enoxaparin followed by warfarin or acenocoumarin (EINSTEIN DVT, EINSTEIN PE)^14,15^ with CAT subset data reported in pooled analysis (EINSTEIN DVT/PE)^28^; two RCTs reported effects of dabigatran compared to LMWH followed by warfarin (RE-COVER I, RE-COVER II)^17,18^ with CAT subset data reported in pooled analysis (RE-COVER I&II)^29^; one RCT reported the effects of edoxaban compared to warfarin (Hokusai 2013)^15^; one RCT reported the effects of edoxaban compared to dalteparin (Hokusai 2018)^19^; one RCT reported the effects of rivaroxaban compared to dalteparin (SELECT-D)^20^; two RCTs reported the effects of apixaban compared to dalteparin (ADAM VTE and Caravaggio)^21,30^. Characteristics of RCT and participants are described in Table 1. Inclusion criteria for individual studies are noted in Fig. 1.

**Table 1:** Description of study and participants. NR, not reported. *type of cancer diagnosis was only reported for 114 patients.

Clinical trial	% cancer patients in study	RCT design	Follow up	Primary outcome	Study participants		
					Characteristic	DOAC	Comparator
ADAM VTE	100%	Open label, superiority	6 months	Major bleeding	Drug Number patients Age, gender Most common cancer Metastatic cancer Anticancer therapy	Apixaban 150 64, 48% male Lung 22% (32/150) 65% (96/150) 73% (108/150)	Dalteparin 150 64, 49% male Pancreatic 16% (24/150) 66% (97/150) 74% (110/150)
Hokusai 2018	100%	Open label, non-inferiority	12 months	VTE recurrence	Drug Number patients Age, gender Most common cancer Metastatic cancer Anticancer therapy	Edoxaban 522 64, 53% male Colorectal (16%) 53% (274/522) 72% (374/522)	Dalteparin 524 64, 50% male Colorectal (15%) 53% (280/524) 73% (383/524)
SELECT-D	100%	Open label, pilot trial	6 months	VTE recurrence	Drug Number patients Age, gender Most common cancer Metastatic cancer Anticancer therapy	Rivaroxaban 203 67, 57% male Colorectal 27% (55/203) 58% (118/203) 69% (140/203)	Dalteparin 203 67, 48% male Colorectal 23% (47/203) 58% (118/203) 70% (142/203)
Caravaggio	100%	Open label, non-inferiority	6 months	VTE recurrence	Drug Number patients Age, gender Most common cancer Metastatic cancer Anticancer therapy	Apixaban 576 67, 51% male Colorectal 21% (121/576) 68% (389/576) 61% (350/576)	Dalteparin 579 67, 48% male Colorectal 19% (113/579) 69% (396/579) 63% (350/579)
Amplify	2.5%	Double- blind, non-inferiority	6 months	VTE recurrence	Drug Number patients Age, gender Most common cancer Metastatic cancer Anticancer therapy	Apixaban 88 66, 57% male Prostate %NR “approximately 1/3” NR	Warfarin 81 65, 61% male Prostate %NR “approximately 1/3” NR
Hokusai 2013	2.5%	Double-blind, non-inferiority	12 months	VTE recurrence	Drug Number patients Age, gender Most common cancer Metastatic cancer Anticancer therapy	Edoxaban 109 NR NR NR NR	Warfarin 99 NR NR NR NR
RE-COVER I & II	7.0%	Double-blind, double-dummy, non-inferiority	6 months	VTE recurrence	Drug Number patients Age, gender Most common cancer Metastatic cancer Anticancer therapy	Dabigatran 173 63, 64% male Prostate 20% (23/114)* 9% (11/114)* NR	Warfarin 162 65, 62% male Prostate 21% (22/107)* 16% (17/107)* NR
EINSTEIN DVT/PE	7.2%	Open label, non-inferiority	3–12 months	VTE recurrence	Drug Number patients Age, gender Most common cancer Metastatic cancer Anticancer therapy	Rivaroxaban 316 NR NR NR NR	Warfarin or acenocoumerol 281 NR NR NR NR

### Risk of bias

**Figure 2:**
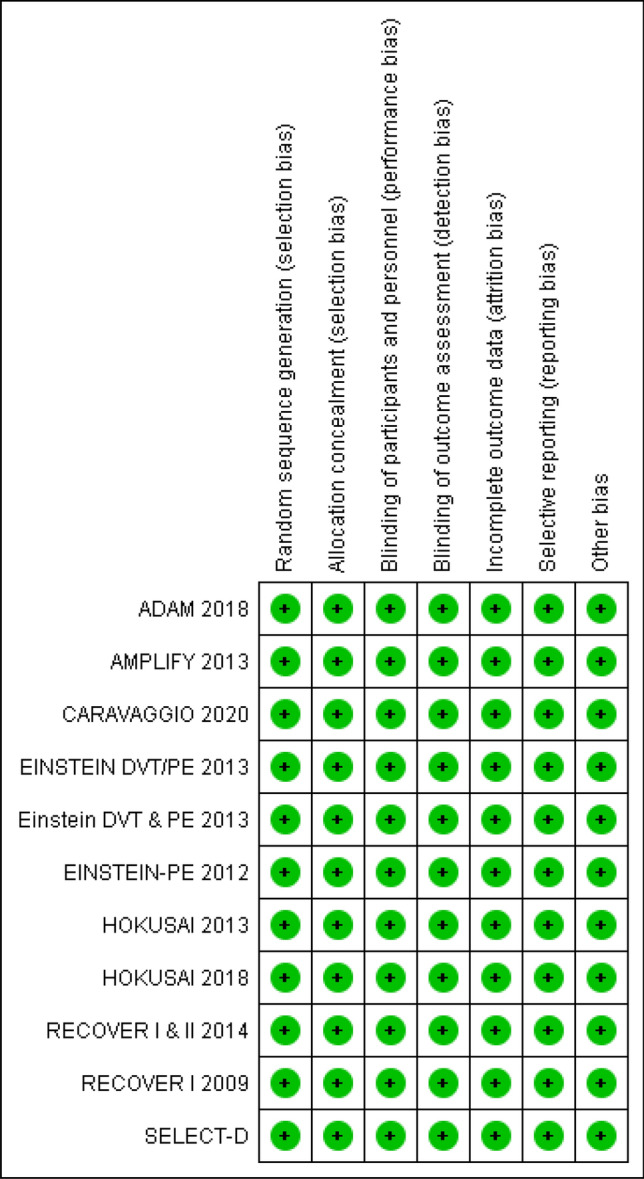
Cochrane Collaboration risk of bias summary.

Overall, included RCTs were free from any major risk of bias (Fig. 2).

### Blinding

Four of ten RCTs were randomized, double blinded clinical trials (Amplify, Re-cover I, Re-cover II, Hokusai 2013). Six of ten RCTs were randomized, open label clinical trials (Einstein DVT, Einstein PE, Hokusai 2018, Select-D, Adam VTE, Caravaggio). All RCTs reported detailed blinding procedures for an independent adjudication process for outcomes assessment. Therefore, all ten RCTs were determined to have low risk for performance and selection bias.

### Outcomes data

Five of ten RCTs reported the intention to treat (ITT) method to evaluate the benefit outcome of recurrent VTE (AMPLIFY, EINSTEN DVT, Einstein PE, SELECT-D, Caravaggio) and utilized per-protocol method to analyze the secondary safety outcomes (bleeding). Accordingly, these four RCTs were assessed to have low risk of attrition bias. Five studies used a modified ITT (mITT) analysis of benefit outcome of recurrent VTE. The RE-COVER I, RE-COVER II, Hokusai 2013, Hokusai 2018, and ADAM VTE defined mITT as population of patients who were randomized and received at least one dose of study drug. The safety outcomes in these RCTs were not analyzed per protocol prespecified rules, instead they were analyzed using mITT population. However, overall difference in analyzed participants between treatment arms were minimal in these studies. Hence these RCTs were also assessed to have low risk of attrition bias.

### Other potential bias

All ten RCTs reported methods of randomization, allocation concealment, and consistently reported the outcomes states before the trial. Pre-specified alpha and beta errors (elements for calculating risk of random error) and sample sizes were reported for all RCTs. All RCTs were therefore assessed as having low risk of selection bias.

### Effects of interventions

Ten RCTs were evaluated for overall effect. Four RCTs (ADAM VTE, Hokusai 2018, SELECT-D, Caravaggio) were included for subgroup analysis comparing DOACs to LMWH and six RCTs (AMPLIFY, EINSTEIN DVT/PE, RE-COVER I & II, and Hokusai 2013) were included for subgroup analysis comparing DOACs to VKA. The Hokusai 2013 study did not report separate safety outcomes for MB or CRNMB and thus was excluded from subgroup analysis of these outcomes.

### Efficacy of DOACs (recurrent VTE)

**Figure 3:**
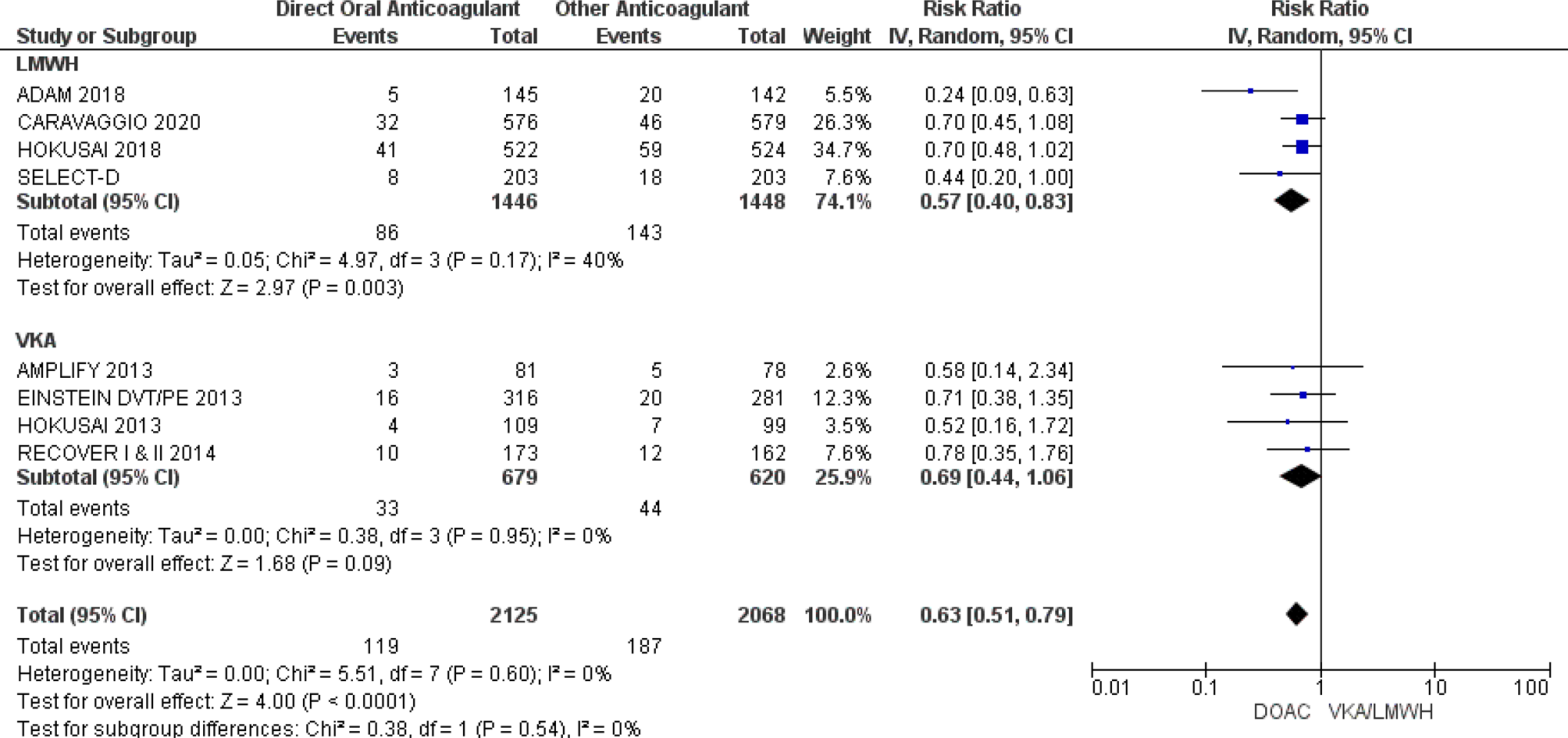
Efficacy (VTE recurrence) of DOAC. Forest plots show risk ratio (RR) of VTE recurrence of pooled data from all studies and subgroup analyses of studies evaluating DOAC compared to LMWH and DOAC compared VKA. Boxes superimposing RR estimates are proportional to the weight of the included study. Heterogeneity between RCT is assessed by the I^2^ statistic.

Data from 4193 participants was pooled for evaluation of recurrent VTE. Participants treated DOACs had lower risk of recurrent VTE compared to participants treated with either VKA or LMWH (RR 0.63; 95% CI 0.51–0.79; p < 0.0001; I^2^ = 0%). In subgroup analysis comparing DOAC to LMWH reduced risk for recurrent VTE was noted with use of DOACs (RR 0.57; 95% CI 0.40–0.83; p = 0.003; I^2^ = 40%). Subgroup analysis comparing DOAC to VKA showed no difference in recurrent VTE risk (RR 0.69; 95% CI 0.44–1.06; p = 0.09; I^2^ = 0%) (Fig. 3). Per GRADE criteria, the quality of evidence was judged to be high for VTE recurrence (Table 2).

**Table 2:** GRADE analysis for quality of evidence.

Outcome	Certainty Assessment							Patients		Anticipated effects		Certainty
	Number studies	Study design	Risk of bias	Inconsistency	Indirectness	Imprecision	Other	DOAC	Other AC	Relative risk (95% CI)	Absolute risk reduction with DOAC (95% CI)	
**DOAC compared to VKA/LMWH**												
VTE	10	RCT	Not serious	Not serious	Not serious	Not serious	None	5.6% (119/2125)	9.0% (187/2068)	0.63 (0.51–0.79)	33 fewer per 1000 (44 fewer to 19 fewer)	⊕ ⊕ ⊕ ⊕ High
MB	9	RCT	Not serious	Not serious	Not serious	Not serious	None	4.3% (86/2008)	4.0% (78/1958)	1.01 (0.65–1.56)	0 fewer per 1000 (14 fewer to 22 more)	⊕ ⊕ ⊕ ⊕ High
CRNMB	9	RCT	Not serious	Not serious	Not serious	Not serious	None	11.7% (235/2008)	8.9% (174/1958)	1.28 (0.95–1.73)	25 more per 1000 (4 fewer to 65 more)	⊕ ⊕ ⊕ ⊕ High
All bleed	10	RCT	Not serious	Not serious	Not serious	Not serious	None	16.1% (341/2117)	13.5% (277/2057)	1.11 (0.84–1.46)	15 more per 1000 (22 fewer to 62 more)	⊕ ⊕ ⊕ ⊕ High
**DOAC compared to LMWH**												
VTE	4	RCT	Not serious	Not serious	Not serious	Not serious	None	5.9% (86/1446)	9.9% (143/1448)	0.57 (0.40–0.83)	42 fewer per 1000 (59 fewer to 17 fewer)	⊕ ⊕ ⊕ ⊕ High
MB	4	RCT	Not serious	Not serious	Not serious	Not serious	None	4.8% (69/1446)	3.7% (53/1448)	1.31 (0.78–2.18)	11 more per 1000 (8 fewer to 43 more)	⊕ ⊕ ⊕ ⊕ High
CRNMB	4	RCT	Not serious	Not serious	Not serious	Not serious	None	11.2% (162/1446)	7.3% (106/1448)	1.6 (1.13–2.26)	44 more per 1000 (10 more to 92 more)	⊕ ⊕ ⊕ ⊕ High
All bleed	4	RCT	Not serious	Not serious	Not serious	Not serious	None	16.0% (231/1446)	11.0% (159/1448)	1.49 (1.1–2.01)	54 more per 1000 (11 more to 111 more)	⊕ ⊕ ⊕ ⊕ High
**DOAC compared to VKA**												
VTE	6	RCT	Not serious	Not serious	Not serious	Not serious	None	4.9% (33/679)	7.1% (44/620)	0.69 (0.44– 1.06)	22 fewer per 1000 (40 fewer to 4 more)	⊕ ⊕ ⊕ ⊕ High
MB	5	RCT	Not serious	Not serious	Not serious	Not serious	None	3.0% (17/562)	4.9% (25/510)	0.62 (0.34– 1.14)	19 fewer per 1000 (32 fewer to 7 more)	⊕ ⊕ ⊕ ⊕ High
CRNMB	5	RCT	Not serious	Not serious	Not serious	Not serious	None	13.0% (73/562)	13.3% (68/510)	0.95 (0.63– 1.41)	7 fewer per 1000 (49 fewer to 55 more)	⊕ ⊕ ⊕ ⊕ High
All bleed	6	RCT	Not serious	Not serious	Not serious	Not serious	None	16.4% (110/671)	19.4% (118/609)	0.84 (0.65– 1.08)	31 fewer per 1000 (68 fewer to 16 more)	⊕ ⊕ ⊕ ⊕ High

*VTE* (recurrent) venous thromboembolism, *MB* major bleeding, *CRNMB* clinically relevant non major bleeding, *all bleeding* MB + CRNMB, *DOAC* direct oral anticoagulant, *other AC* other anticoagulant (VKA or LMWH).

### Safety outcomes (bleeding)

**Figure 4:**
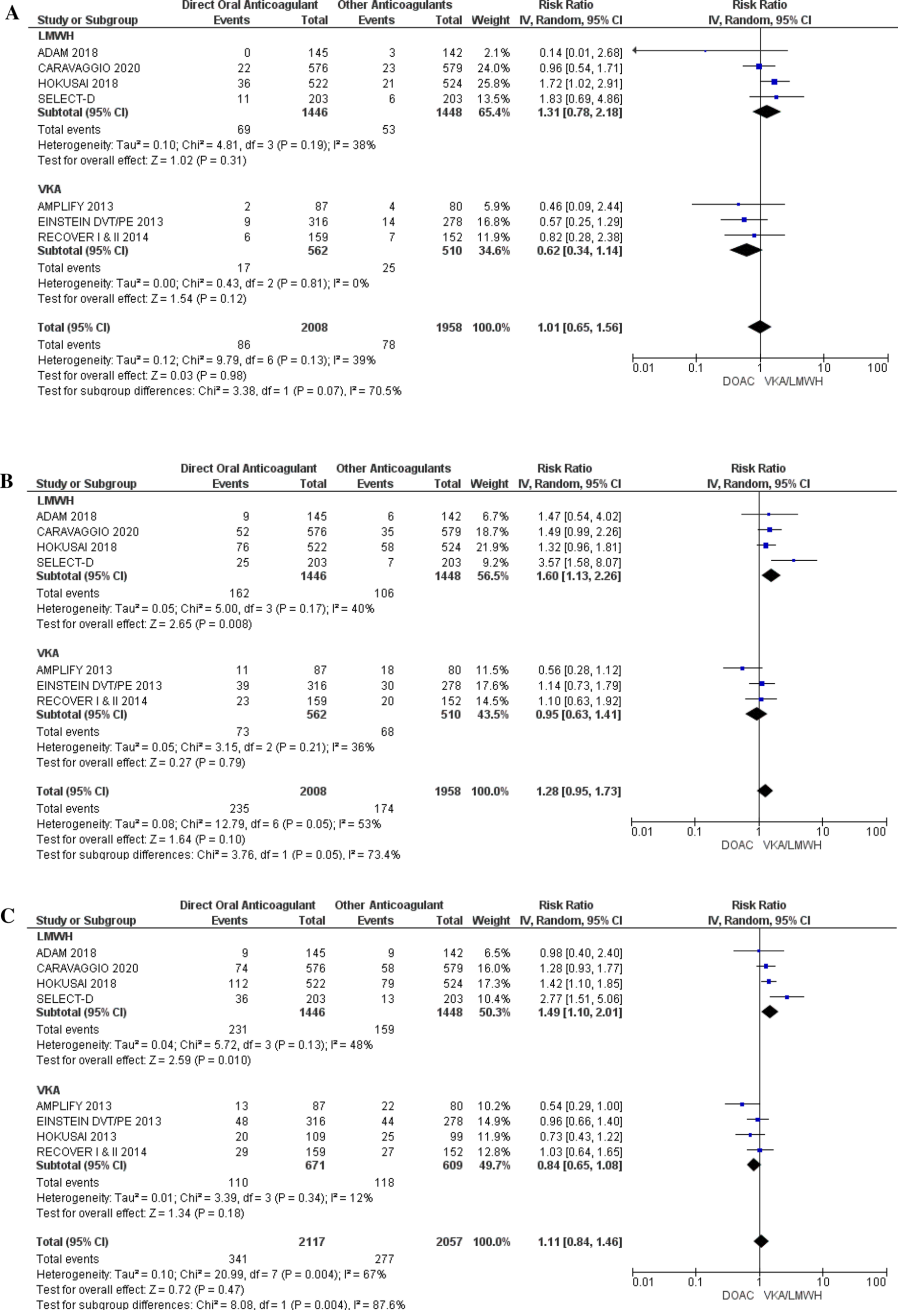
Safety (bleeding risk) of DOAC. **(a)** Major bleeding (MB), **(b)** clinically relevant non major bleeding (CRNMB), **(c)** all bleeding (composite MB and CRNMB). Forest plots show risk ratio (RR) of VTE recurrence of pooled data from all studies and subgroup analyses of studies evaluating DOAC compared to LMWH and DOAC compared to VKA. Boxes superimposing RR estimates are proportional to the weight of the included study. Heterogeneity between RCT is assessed by the I^2^ statistic.

Data for MB and CRNMB were extracted from nine RCTs (n = 3966 participants) and data for all bleeding (major bleeding + CRNMB) were extracted from ten RCTs (n = 4147 participants). Overall, no difference in risk of MB (RR 1.01; 95% CI 0.65–1.56; p = 0.98; I^2^ = 39%), CRNMB (RR 1.28; 95% CI 0.95–1.73; p = 0.10; I^2^ = 53%), or all bleeding (RR 1.1; 95% CI 0.84–1.46; p = 0.47; I^2^ = 67%), was observed in patients treated with DOACs versus comparators. Compared to LMWH, DOACS had no difference in MB risk (RR 1.31; 95% CI 0.78–2.18; p = 0.31; I^2^ = 38%) was observed but had increased risk for CRNMB (RR 1.60; 95% CI 1.13–2.26; p = 0.008; I^2^ = 40%) and all bleeding (RR 1.49; 95% CI 1.10–2.01; p = 0.010; I^2^ = 48%). Compared to VKA, DOACS showed no difference in MB (RR 0.62; 95% CI 0.34–1.14; p = 0.12; I^2^ = 0%), CRNMB (RR 0.95; 95% CI 0.63–1.41; p = 0.78; I^2^ = 36%), and all bleeding (RR 0.84; 95% CI 0.81–3.01; p = 0.18; I^2^ = 27%) risks (Fig. 4). Per GRADE criteria, the quality of evidence was judged to be high for safety outcomes (Table 2).

## Discussion

The purpose of our meta-analysis is to evaluate the efficacy and safety of DOACs for the treatment of CAT. Incorporating 4193 participant data our meta-analysis is an update on prior studies on the same subject.

The efficacy results of our meta-analysis indicate that DOACs are superior to VKA or LMWH for secondary prevention of VTE in patients with CAT. The improved efficacy with DOACs is demonstrated in comparison to LMWH, the current standard of care for CAT, but is not demonstrated compared to VKA. However, it should be noted that while statistically important, the benefit of DOACs is small, with absolute risk reduction of 3.3% overall and 4.2% compared to LMWH. The safety results of our meta-analysis indicate no difference in MB, CRNMB, or all bleeding risk with DOACs overall, but note risk of CNRMB and all bleeding without increased MB risk compared to LMWH. However, absolute risk increases with DOACs compared to LMWH is small for both CRNMB (4.4%) and (5.4%). No difference in bleeding risk with DOACs compared to VKA is noted. These results indicate that DOACs are efficacious drugs for treatment of CAT though have an increased risk for non-major bleeding compared to LMWH.

Previous, smaller studies on the same topic have noted variable efficacy and bleeding risks with DOACs. In one meta-analysis of 2151 participants including the Hokusai 2018 study, one study reported reduced risk for VTE recurrence in patients treated with DOACs compared to LMWH or VKA (RR 0.64; 95% CI 0.46–0.88) but no difference in bleeding outcomes^31^. Another meta-analysis of 1952 participants from nine RCTs with unselected cancer subpopulations noted similarly reduced VTE recurrence risk with DOACs compared to LMWH or VKA though there was no difference in VTE recurrence rate and bleeding risk in subgroup analysis of two RCTs comparing DOACs to LMWH^32^. A meta-analysis including 1132 participants’ from six RCTs with unselected cancer subpopulations comparing DOACs to VKA showed no difference in risk for VTE recurrence, MB, or CRNMB with DOACs^33^. A meta-analysis of two RCT comparing DOACs to LMWH (Hokusai 2018 and SELECT-D) no difference in VTE recurrence risk was observed but an increased risk for MB was noted (RR 1.74; 95% CI 1.05–2.88) with DOACs^34^. Most recently, Brunetti et al. showed superiority of DOACs over LMWH in meta-analyses including ADAM VTE, Hokusai 2018, and SELECT-D studies (RR 0.56; 95% CI 0.40–0.79)^35^. With the addition of the Caravaggio trial data, the results of our meta-analysis are only the second to show superiority for secondary VTE prevention with DOACs and the first to note no increase in MB with DOACs overall or compared to LMWH.

One explanation for the favorable efficacy with DOACs compared to LMWH is compliance with treatment. In general, compliance and patient desire to take drug is higher with DOACs, leading to longer on treatment time, than with LMWH. In the Hokusai 2018 study, premature discontinuation of study drug due to “patient decision” occurred in 15% of patients treated with dalteparin compared to 4% treated with edoxaban^19^. In the SELECT-D study, 10% of patients treated with dalteparin discontinued treatment due to either “patient decision” or “withdrawal of consent by the patient” compared to 6% in the rivaroxaban group^20^. In the ADAM VTE study, 15% of patients in the dalteparin group refused further treatment compared to 4% in the apixaban group^30^. Though compliance with DOAC and LMWH was roughly the same, the Caravaggio study reported withdrawal of consent as cause for permanent study drug discontinuation in 16% of patients in the dalteparin group, compared to 5.8% of the apixaban group^21^. Increased adherence and on treatment time with DOACs has also been noted in real world data. In an insurance claims database study, longer average on-treatment time with rivaroxaban than LMWH (3 months vs. 1 month) was noted, suggesting noncompliance and/or suboptimal treatment is prevalent with LMWH in clinical practice^36^.

The results of our meta-analysis that depicts comparable efficacy of DOACs and VKA for secondary prevention of CAT has several limitations. Firstly, large confidence intervals are noted on individual studies and in the pooled results of our meta-analysis, suggestive of underlying uncertainty in precision of risk ratio. This is likely reflective of small sample size of patients with active malignancy within each RCT populations (3–7% of trial population). In addition, there is notable exclusion of patients with aggressive cancers (patients with end organ dysfunction, reduced life expectancy, low percentage of metastatic cancers, and low percentage of patients on chemotherapy) which may have limited representation of patients at highest risk for VTE and recurrence of VTE. In fact, VTE recurrence with VKA in our meta-analysis showed a pooled rate ~ 7% (44/620) which is lower than the rate of VTE with VKA (10–15%) in the CATCH and CLOT trials which evaluated VKA for CAT treatment in a population of cancer patients^9,37^. Nevertheless, though statistically not different, we note numerically fewer VTE recurrences in patients treated with DOACs. Differences in efficacy between DOACs and VKA for treatment of CAT could be better evaluated in a dedicated randomized study with cancer patients, but this type of study is of low clinical value as LMWH has been clearly shown to be superior to VKA for treatment of CAT^9,37,38^.

Interpretation of bleeding risks associated with DOACs is complex. Statistically, no significant heterogeneity exists between studies in subgroups comparing DOACs to VKA or DOACs to LMWH, lending reassurance about cross trial comparison in subgroup analysis. However, significant heterogeneity does exist with regards to the overall meta-analysis and is likely a result of significant differences in trials, including study definitions of bleeding, active malignancy, participant inclusion and exclusion criteria, and sample selection. Thus, interpretation of overall bleeding results is limited and likely not clinically useful and bleeding risk conclusions may best be characterized in subgroup analysis.

The etiology of favorable MB profile, increased CRNMB, and all bleeding risk described with DOACs compared to LMWH is unclear, though increased bleeding with DOACs seems to be related to mucosal bleeding. Factors influencing bleeding include malignancy type and DOAC type with higher risk observed in upper gastrointestinal malignancies and with use of edoxaban and rivaroxaban. In the Hokusai 2018 study, a significant difference in MB was noted. However, when stratified by cancer type, only gastrointestinal malignancies showed increased MB rates with edoxaban compared to dalteparin (13.2% vs. 2.4%)^19^. Similarly, in the SELECT-D study, patients with esophageal or gastroesophageal junction cancers were excluded from enrollment after interim analysis showed three-fold increase in major bleeding with rivaroxaban in these cancer types. CRNMB was statistically higher in patients treated with rivaroxaban in the SELECT-D study, with most prevalent bleeding cancers being bladder and colorectal and most prevalent bleeding sites being genitourinary and gastrointestinal^20^. The risk for bleeding in patients with gastrointestinal cancers was also noted in a large retrospective cohort study with a MB rate of 16.7% with rivaroxaban compared to 7.2% with LMWH^39^. Conversely, the ADAM VTE trial, which was powered to detect bleeding differences and included patients with both upper and lower gastrointestinal malignancies, did not show increased risk for MB or CRNMB in patients treated with apixaban. However, these data are difficult to interpret as the study did not meet its prespecified primary outcome due to lower than anticipated bleeding rates for both treatment arms. The Caravaggio study, which included patients with different malignancies including gastrointestinal cancers in similar proportions to the Hokusai 2018 study, did not show differences in MB or CRNMB between apixaban and LMWH treated patients. Bleeding data from the Caravaggio study was not stratified by cancer type. No difference in bleeding site including gastrointestinal bleeding was noted for MB in the Caravaggio study. Interestingly, the Caravaggio study did note greater numbers of CRNMB with apixaban compared to dalteparin (52/576 vs. 34/579) with most prevalent sites of bleeding being genitourinary, upper airway, and gastrointestinal, which suggests possible systemic mucosal bleeding risk instead of localized bleeding risk from the tumor itself.

Ultimately, many of the observed patterns of bleeding with DOACs may be related to intrinsic properties of DOACs themselves. Several mechanisms have been conjectured to explain gastrointestinal bleeding with specific DOACS including topical anticoagulant effects (dabigatran, rivaroxaban, edoxaban), direct caustic effect (tartaric acid in dabigatran), and pharmacodynamic differences (higher peak concentrations with rivaroxaban and edoxaban)^40^. Currently, there are no head-to-head comparison studies evaluating bleeding risks with different DOACs. However in one meta-analysis of 43 trials utilizing DOACs for any indication including VTE (but excluding CAT), increased gastrointestinal and CRNMB risks were noted in patients treated with dabigatran and rivaroxaban but not with apixaban^41^. In another meta-analysis of 28 observational database studies of DOACs use in atrial fibrillation, dabigatran and rivaroxaban had increased risk and apixaban had lower risk for gastrointestinal bleeding and major hemorrhage compared to LMWH or VKA^42^. There is not enough data to perform a subgroup meta-analysis of bleeding risk, stratified by cancer type and DOAC type to confirm these observations; hence, these results warrant further study with a clinical trial.

Though our results show equivalence of bleeding risk with DOACs and VKA, the interpretation of these results is limited by uncertainty of precision with small sample sizes and exclusion of high bleeding risk cancer types. Thus, our results are likely not truly reflective of risks of bleeding with DOACs compared to VKA. We presume VKA has similar bleeding risk as LMWH in treatment of CAT, as previously noted in RCT incorporating patients with high risk for bleeding and in meta-analyses^9,37,38,43^. The incidence of bleeding with VKA in our study is approximately four times lower than LMWH in our meta-analysis, suggesting exclusion of high bleeding risk cancer patients in the AMPLIFY, RE-COVER I & II, EINSTEIN DVT/PE, and Hokusai 2013 studies.

A strength of our meta-analysis is the inclusion of all current high quality RCTs evaluating currently available DOACs for treatment of CAT, thus providing strength to outcomes findings, as evidenced by the GRADE analysis showing high certainty for outcomes. In addition, the results of our meta-analysis are generally similar to real-world observational studies showing benefit of DOACs in CAT treatment without major increase in bleeding^36,44,45^. These studies generally incorporate more variability in patient population, including presence of high VTE risk cancer subtypes, cancer severity (stage), access to care, duration of treatment, and patient comorbidities, and thus lend generalizability and clinical relevance to our findings.

There are limitations to our meta-analysis and the results should be included in the context of the DOACs included. Our results are heavily influenced by the outcomes of the Caravaggio and Hokusai 2018 studies which enrolled more than half of all participants and more than three fourths of participants in subgroup analysis comparing DOACs to LMWH. Thus, edoxaban and apixaban are over-represented as DOAC types in this meta-analysis and are primarily the drivers of outcomes. Additionally, no direct comparison of dabigatran to LMWH for treatment of CAT exists and < 10% of patients in the study received dabigatran overall. Therefore, it remains unclear whether the results of this meta-analysis can be extrapolated to dabigatran. Furthermore, while data comparing DOACs to LMWH are clinically relevant, the findings of equivalence of DOACs to VKA is not clinically useful, as LMWH has been previously shown to be superior to VKA for treatment of CAT^38,43^. Few patients with hematologic malignancies or hematopoietic stem cell transplant were included in the studies and it remains unclear whether the results noted here can be applied to such patients. Lastly, the duration of anticoagulation in patients with CAT is not clear. Most studies in this meta-analysis provided anticoagulation for 6–12 months, but many patients have ongoing risk factors for thrombosis for an extended duration of time.

The CANVAS study (NCT 02744092), evaluating the role of DOAC (rivaroxaban, apixaban, edoxaban, or dabigatran), is currently ongoing and should help clarify the role of DOACs and specifically provide more data regarding use of dabigatran, in the management of CAT. The EVE Trial (NCT 030808), evaluating extended duration apixaban in CAT is currently ongoing and will provide more evidence about the duration and dosing of anticoagulation in patients with CAT.

## Conclusion

This meta-analysis shows that DOACs are more effective at preventing VTE recurrence though carry a small risk for increased non-major bleeding. In the appropriately selected patient, DOACs are safe for the treatment of CAT. The results warrant consideration for changing the paradigm for treatment of CAT.
